# Analyzing the impact of glaucoma on the macular architecture using spectral-domain optical coherence tomography

**DOI:** 10.1371/journal.pone.0209610

**Published:** 2018-12-31

**Authors:** Jan D. Unterlauft, Matus Rehak, Michael R. R. Böhm, Franziska G. Rauscher

**Affiliations:** 1 Department of Ophthalmology, Leipzig University Hospital, Leipzig, Germany; 2 Department of Ophthalmology, University Hospital Essen, University of Duisburg/Essen, Essen, Germany; 3 Institute for Medical Informatics, Statistics and Epidemiology, Leipzig University, Leipzig, Germany; Massachusetts Eye & Ear Infirmary, Harvard Medical School, UNITED STATES

## Abstract

**Purpose:**

Using spectral domain optical coherence tomography (SD-OCT) the retina can be segmented automatically to visualize all retinal layers. In glaucoma chronically elevated intraocular pressure leads to a decline of retinal ganglion cells (RGC) which changes retinal architecture. The goal of these analyses was to gain insight into the changes induced by glaucoma within all macular layers using SD-OCT within a closely circumscribed glaucoma cohort.

**Materials and methods:**

SD-OCT measurements with automated retinal layer segmentation were performed in both eyes of primary open-angle glaucoma patients with a defined monocular absolute visual field scotoma in the central 10° of the visual field and in an age-matched healthy control group. Thickness of single retinal layers and entire retina were compared with special attention to the localization of the visual field scotoma in the glaucomatous eyes.

**Results:**

30 eyes of 15 glaucoma patients and 15 eyes of 15 healthy controls were included in this study. Statistical significant thickness differences were detected in the control group between superior and inferior retina for the retinal nerve fiber layer (RNFL), the outer plexiform layer (OPL) and the outer nuclear layer (ONL). In the glaucoma group thickness differences between worse and less affected eyes in the RNFL, the ganglion cell layer (GCL) and the inner plexiform layers (INL) were found. Comparison between healthy and diseased eyes revealed significant thickness differences in the RNFL, GCL, IPL and total retinal thickness but not the outer retinal layers.

**Conclusion:**

Comparison between SD-OCT measurements of the macula between healthy and glaucomatous eyes in a closely circumscribed disease stage showed a pronounced disease impact on the inner but not the outer retina. These results provide evidence that GCL and IPL thickness seem to be good measures to discriminate between affected and unaffected eyes in testing for glaucoma.

## Introduction

Much is known about apoptotic retinal ganglion cell (RGC) loss and the resulting optic nerve head atrophy in glaucoma which leads to visual field defects and can ultimately result in blindness [1–3]. In advanced disease stages, optic nerve head atrophy can be visualized easily by indirect ophthalmoscopy at the slit lamp alone [4–6]. Early detection leads to earlier treatment start, saving patients from developing irrecoverable damage. Since most forms of glaucoma develop continuously over years before becoming clinically apparent as loss of retinal nerve fiber layer (RNFL) and visual field scotomas, there is a necessity for early assessment paradigms aiding clinical diagnosis [7]. Optical coherence tomography (OCT) techniques measure thickness decrease of the ganglion cell layer (GCL) and the RNFL in the macular region and at the optic nerve head [8–10]. Increasing OCT image resolution makes it feasible to measure the thickness of all retinal layers as well as monitoring their changes over time. Using spectral domain (SD)-OCT the thickness of up to eight different retinal layers can be determined with good repeatability and reproducibility [11]. Using these techniques the thickness of single retinal layers can be measured in the peripapillary region as well as in the macula and fovea regions [12]. Previous studies have reported glaucomatous damage resulting in decreased RNFL, GCL and inner plexiform layer (IPL) thickness but no changes in outer retinal layer thicknesses [13, 14].

It is well established that loss of retinal ganglion cells in glaucoma leads to apparent changes of the optic nerve head but also presents with widespread changes of the central nervous system and the ascending visual pathways [15, 16]. RGC demise could therefore in theory also lead to loss of further downstream connected neural cell layers. Recent studies reported photoreceptor swelling and other abnormalities of the outer retina indicating primary loss of photoreceptors in glaucoma [17–21]. This has been demonstrated using ultra-high resolution and adaptive optics OCT scans [22–24].

The aim of this study was to analyze the thickness of single neuroretinal layers of the central retina including the macula of glaucoma patients in advanced disease stages using SD-OCT in comparison to an age-matched group of healthy subjects. Furthermore, we wanted to elucidate whether all retinal layers are equally sensitive to glaucomatous impact in advanced disease stages. Finally, we sought to investigate whether in glaucoma anatomic changes are not only present downstream but also upstream from the retinal ganglion cells, a finding which would emphasize the neurodegenerative nature of this disease.

## Materials & methods

For this study we included patients with open angle glaucoma in advanced disease stages who were referred to our clinic for further treatment between July and December 2017. Advanced disease stage was defined as a present circumscribed absolute visual field scotoma in one eye and presence of apparent glaucomatous optic nerve head atrophy upon fundus examination. To verify these and the glaucoma diagnosis a complete ophthalmological examination was performed. This included history taking, best corrected visual acuity (BCVA) testing using Snellen charts (transformed to logMAR for statistical analysis), slit lamp examination of the anterior and posterior segments, evaluation of the optic nerve head by indirect ophthalmoscopy, static automatic visual field testing (Twinfield 2, Oculus, Wetzlar, Germany; 24–2 test strategy, 55 target positions with 16 target points falling into the central 10° of the visual field) and Goldmann applanation tonometry.

**Fig 1:**
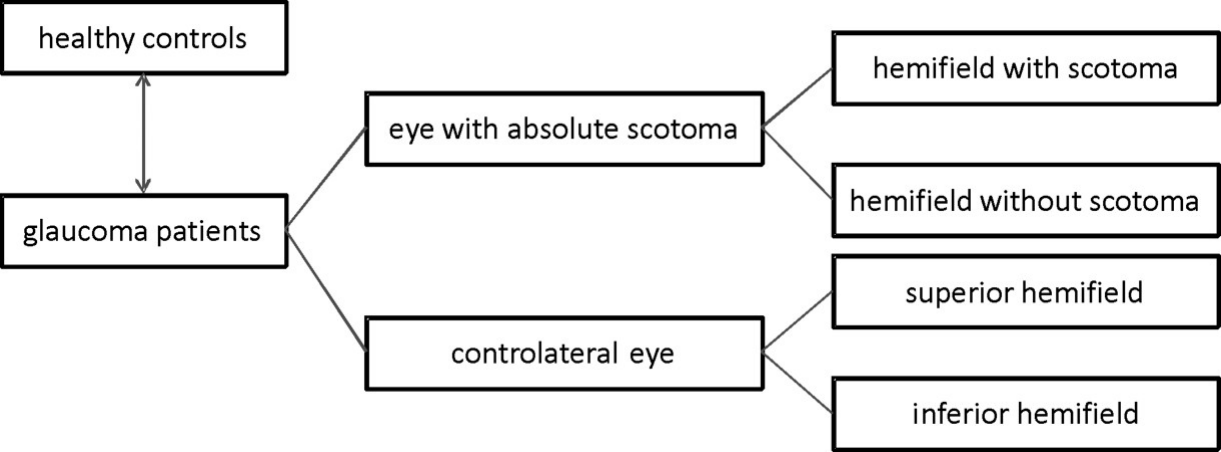
Grouping of patients, eyes and hemifields included into the analyses. Presentation of grouping for analysis of macular retinal layers in glaucoma eyes and healthy controls.

Glaucoma patients had to meet the following criteria to be included into the analysis: BCVA ≥ 0.1 logMAR, deep anterior chamber with open anterior chamber angle, regulated intraocular pressure between 10 and 21 mmHg, reliable visual field testing (false negatives and false positives ≤10%), absence of other ophthalmic or neurologic diseases causing visual field defects other than glaucoma and absence of apparent pathological changes of the macula due to age-related macular degeneration, diabetic maculopathy, epiretinal membrane formation or any other. The present absolute visual field scotoma had to be circumscribed and had to be restricted to one hemifield above or below the horizontal within the central 10° of only one eye (Fig 1). The absolute visual field scotoma was defined as a visual field location of strongly reduced light sensitivity of 0 dB. Visual field criteria were strictly defined for consistency and better comparison of OCT images. Subjects included into the control group had to be without any signs for a present ophthalmic disease in the anterior and posterior segments, a BCVA ≥ 20/25 had to be reached, and the refraction of worn glasses had to lie within a spherical equivalent between -5.0 and +5.0 diopters. Visual field testing had to show the absence of any scotoma.

OCT-measurements were obtained by SPECTRALIS SD-OCT (Heidelberg Engineering, Heidelberg, Germany) which uses a confocal scanning laser ophthalmoscope capturing 40,000 A-scans/minute. The axial and transversal resolutions were 4 μm and 6 μm respectively. Active eye tracking was used. 61 parallel B-scans of the macula region with a distance of 0.41° (approximately 120 μm) were performed to capture a central area of 30° (x-direction) x 25° (y-direction). The signal-to-noise ratio was increased by the use of averaging techniques per scanning location. For every B-scan 9 single scans were acquired and averaged to reduce the speckle noise in the images. B-scans were oriented parallel to the line intersecting the foveal center and the Bruch’s membrane opening center which was set by the internal algorithm within the SD-OCT device and checked by the operator before taking the scans for further analysis. A grid of 8 x 8 fields was laid over the macula region to divide this area into 64 evenly distributed arrays. Each grid was 0.83 mm by 0.83 mm in size resulting in a total area of 6.64 mm^2^.

The scan paradigm is partnered with a complex analysis system using the layer segmentation analysis inherent in the SD-OCT machine, which was carried out for all 61 B-scans and interpolated between them. Point-by-point thicknesses were extracted from the scans by subtracting the distance between layer boundaries. The total scan area was divided into 64 (8 x 8) fields of equal size (0.83 mm by 0.83 mm). Point-by-point thicknesses are then averaged within each of these fields. Within these fields, the average layer thicknesses are reported for seven retinal layers and as total retinal thickness.

The 64 macular arrays were scanned and measured in both eyes of a group of 15 glaucoma patients with a single circumscribed absolute visual field scotoma localized in the central 10° and in both eyes of each person in a healthy age-matched comparison group. In the glaucoma patients the visual field test served to localize the absolute visual field defect (see also Fig 1 for further clarification concerning the groups of patients and groups of eyes tested). The SD-OCT scans were then automatically segmented to measure the mean thickness of seven different retinal layers separately analyzed for all 64 arrays. Examined layers were the retinal nerve fiber layer (RNFL), the ganglion cell layer (GCL), inner and outer plexiform layers (IPL & OPL), inner and outer nuclear layers (INL & ONL) and the outer retinal layer (ORL) which contained external limiting membrane, the photoreceptor outer segments and the retinal pigment epithelium. The mean thickness of these layers were measured automatically in each of the 64 arrays of the macular area and taken for further analysis. Each measurement was checked by two independent investigators and segmentation was adjusted manually if necessary.

### Analysis settings

**Fig 2:**
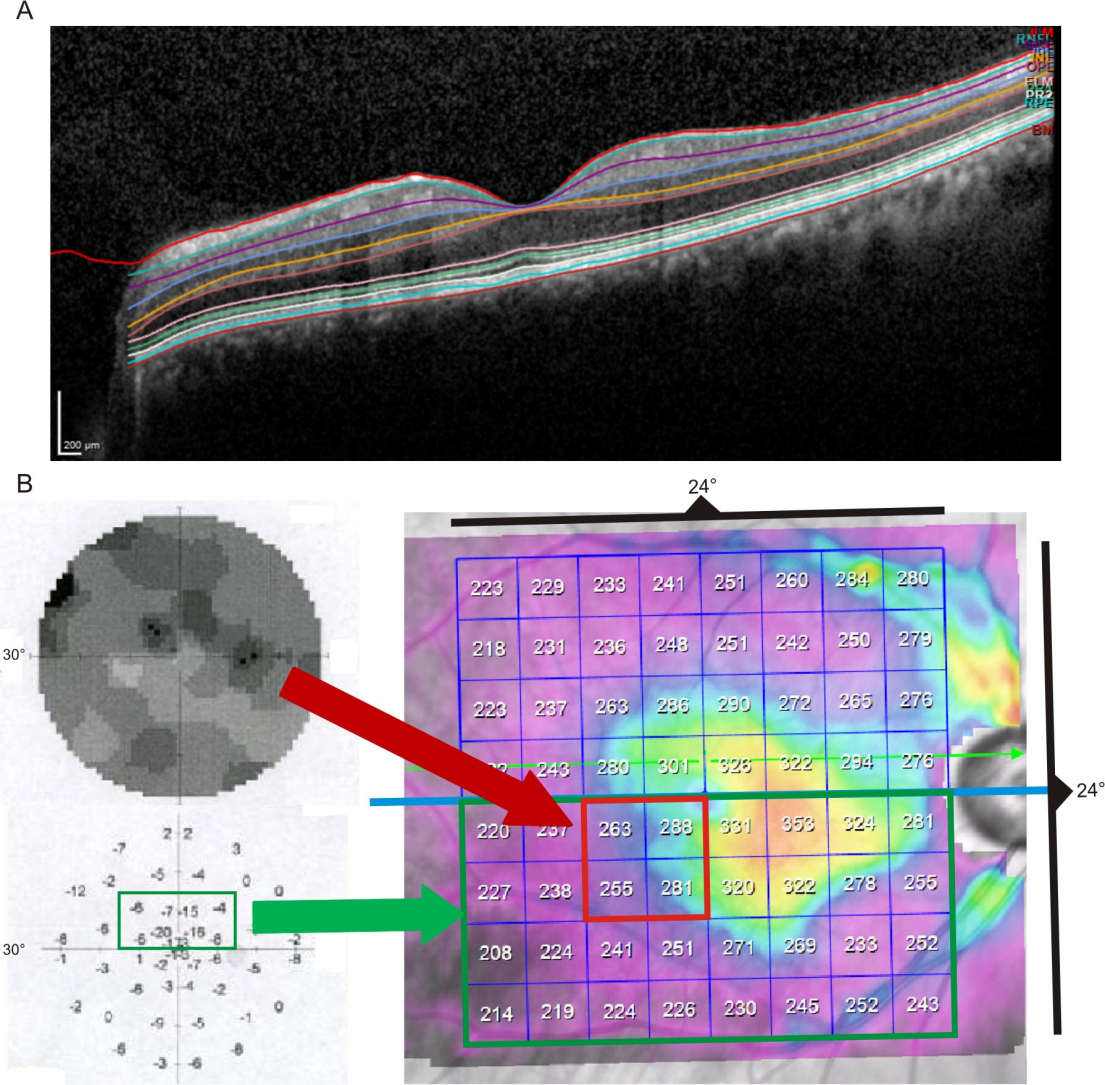
Locations and layering of analyses. A) Single SD-OCT scan in the macular region including foveola and optic nerve head. The automated retinal layer segmentation is outlined in different colors. B) Schematic description of proceeding during hemifield analysis. 30° static automatic perimetry map with an absolute visual field scotoma in the central 10° (red dotted box). The arrays in the SD-OCT-map corresponding to the scotoma are outlined in the 8 x 8 map of the macula. The green dotted box indicates the corresponding visual field sectors with arrays of the inferior hemifields of the SD-OCT scan.

Central to the analyses of the glaucoma patients were existence and localization of a single monocular circumscribed absolute visual field scotoma in the central 10° of the visual field test. To analyze the impact of glaucomatous damage on the central retinal architecture, the localization of the absolute visual field scotoma was matched with the corresponding areas within the SD-OCT-scans of the macula. The mean thickness of each retinal layer and the total retinal thickness were measured in all 64 arrays and further analyzed (termed *hemifield analysis)*. The SD-OCT maps consisting of 64 arrays were divided into hemifields above and below the horizontal line going through the central optic nerve head and the foveola which was set by the SD-OCT device and roughly matches the raphe of the central retina. For each hemifield consisting of 32 arrays the mean thickness of all retinal layers was calculated. The mean layer thickness of the hemifield which contained the corresponding absolute visual field scotoma was then compared to the other hemifield above / below the horizontal dividing line of the same eye (Figs 1 and 2). Further, the mean layer thickness within the hemifield with the absolute visual field scotoma was compared to those of two corresponding hemifields of the contralateral eye related to the position of the scotoma in the upper or lower hemifield (in respect to slight differences of the retinal architecture within the superior and inferior hemifield). Finally, the mean layer thickness in the scotoma hemifield and the corresponding hemifield of the contralateral eye were compared to the equivalent hemifields of the healthy comparison group depending on scotoma localization.

Data collection and statistical analysis were performed using Excel (Version 2007, Microsoft; Redmond, USA) and SPSS (IBM Version 24.0; Chicago, Illinois, USA) software. Patient characteristics, including patient age, BCVA, refraction, visual field indices and retinal layer thickness are given as mean and standard deviation. Non-parametric Wilcoxon-, Mann-Whitney-U- and Friedman-Tests were performed considering a p ≤ 0.05 as indicating statistical significance. Bonferroni correction was applied for multiple testing.

The study adhered to the Tenets of the Declaration of Helsinki and approval for the study was obtained from the Ethics Committee of the Medical Faculty of Leipzig University (209 /18-ek). Written consent was obtained.

## Results

30 eyes of 15 glaucoma patients (3 male; 12 female) with apparent partial optic nerve head atrophy in slit lamp biomicroscopy and repeatedly detectable circumscribed absolute visual field defects in the static automated visual field test were included in this study. Mean patient age was 72.3±8.1 years. Mean BCVA was 0.05±0.06 logMAR (see also Table 1). Mean spherical equivalent was +0.88±1.72 diopters. Mean loss variance (LV) and mean defect (MD) were 33.5±14.4 and 7.9±3.5 dB respectively in the worse affected eye (i.e. the eye with the circumscribed absolute visual field scotoma) in all cases. One eye of each patient showed an absolute visual field defect. In five cases this was the right eye in the remaining ten cases the left eye had the circumscribed absolute visual field defect.

**Table 1:** Characteristics of glaucoma patients and healthy controls included in the analysis of SD-OCT results.

	glaucoma patients	healthy controls	p =
**number [n]**	15	15	
**gender [m : f]**	3 : 12	5 : 10	0.42
**age [years]**	72.3 ± 8.1	66.0 ± 7.6	0.12
**BCVA [logMAR]**	0.05 ± 0.06	0.02 ± 0.04	0.26
**spherical equivalent [D]**	+0.88 ± 1.72	+0.25 ± 1.07	0.32

The control group consisted of 15 eyes of 15 healthy individuals which were age-matched to the group of glaucoma patients enrolled in this analysis. The control group consisted of 5 men and 10 women with a mean age of 66.0±7.6 years. Mean BCVA was 0.02±0.04 logMAR and mean spherical equivalent was +0.25±1.07 diopters. Mean age (p = 0.12), BCVA (p = 0.26) and spherical equivalent of worn spectacles (p = 0.32) did not show a difference of statistical significance in between the glaucoma and the comparison groups.

The mean values for each of the measured seven retinal layers as well as the entire retinal thickness of the control group are summarized in Table 2. Statistical analysis (Wilcoxon-test) revealed differences in the thickness of the RNFL (p<0.01), IPL (p<0.01), ONL (p<0.01), ORL (p<0.01) and complete retinal thickness (p = 0.03) between superior and inferior hemifields in healthy controls. In case of RNFL, IPL, ORL and complete retina the mean thickness of the superior 32 arrays were significantly thinner than the mean of the 32 arrays below the midline connecting foveola and central optic disc. In the healthy subjects the mean inferior ONL thickness was significantly thinner than the mean superior ONL thickness. GCL, INL, OPL and complete retinal thickness did not show significant differences when comparing superior and inferior hemifields.

**Table 2:** Hemifield analysis in the control group. Mean thickness of retinal layers and the entire retina in the macular region in the superior and inferior hemifield above and below the dividing line connecting the foveola and the central optic nerve head. Significant differences between the superior and inferior hemifields were found for the RNFL, IPL, ONL, ORL as well as for the complete retinal thickness.

	superior hemifield	inferior hemifield	p =
**RNFL**	38.2 ± 5.7 μm	46.9 ± 3.9 μm	**<0.01**
**GCL**	30.6 ± 2.4 μm	30.8 ± 2.1 μm	0.93
**IPL**	26.3 ± 1.8 μm	26.7 ± 2.3 μm	**<0.01**
**INL**	29.9 ± 1.9 μm	29.6 ± 3.3 μm	0.98
**OPL**	26.1 ± 2.1 μm	26.7 ± 2.3 μm	0.59
**ONL**	59.6 ± 5.9 μm	54.9 ± 5.2 μm	**<0.01**
**ORL**	78.2 ± 2.4 μm	79.2 ± 2.8 μm	**<0.01**
**entire retinal thickness**	285 ± 19 μm	293 ± 10 μm	**0.03**

Hemifield analysis in the glaucoma eyes showed differences of statistical significance most notably in the RNFL, GCL and IPL of the superior compared to inferior hemifields (Wilcoxon-test). The exact averaged values for the measured thicknesses for the seven retinal layers and the complete retinal thickness for the four hemifields of all glaucoma patients can be taken from Table 3.

**Table 3:** Hemifield analysis in the glaucoma group. Mean thickness of the retinal layers and the entire retina. Results are shown for the hemifields corresponding to the localization of the absolute scotoma in the visual field, the opposite hemifield of the ipsilateral eye, and the superior and inferior hemifield of the contralateral eye.

	scotoma hemifield	ipsilateral remaining hemifield in scotoma eye	corresponding contralateral hemifield to scotoma	Non-corresponding contralateral hemifield	p =
**RNFL**	22.3 ± 2.8 μm	28.2 ± 8.3 μm	36.0 ± 10 μm	34.7 ± 8.9 μm	**<0.01**
**GCL**	21.9 ± 2.1 μm	24.8 ±3.5 μm	28.6 ± 4.1 μm	27.5 ± 4.8 μm	**<0.01**
**IPL**	22.1 ±2.4 μm	23.3 ± 2.2 μm	24.4 ± 2.8 μm	25.1 ± 2.9 μm	**<0.01**
**INL**	32.3 ± 3.4 μm	32.3 ± 3.6 μm	31.3 ± 2.3 μm	31.2 ± 2.5 μm	0.23
**OPL**	26.9 ± 2.4 μm	28.3 ± 2.5 μm	27.1 ± 2.3 μm	28.7 ± 3.0 μm	0.34
**ONL**	55.7 ± 7.8 μm	56.0 ± 7.5 μm	55.2 ± 8.3 μm	56.1 ± 6.8 μm	0.91
**ORL**	79.3 ± 1.9 μm	79.3 ± 2.2 μm	79.2 ± 2.5 μm	78.7 ± 1.9 μm	0.66
**entire retinal thickness**	265 ± 19 μm	274 ± 18 μm	280 ± 21 μm	280 ± 22 μm	**<0.01**

**Fig 3:**
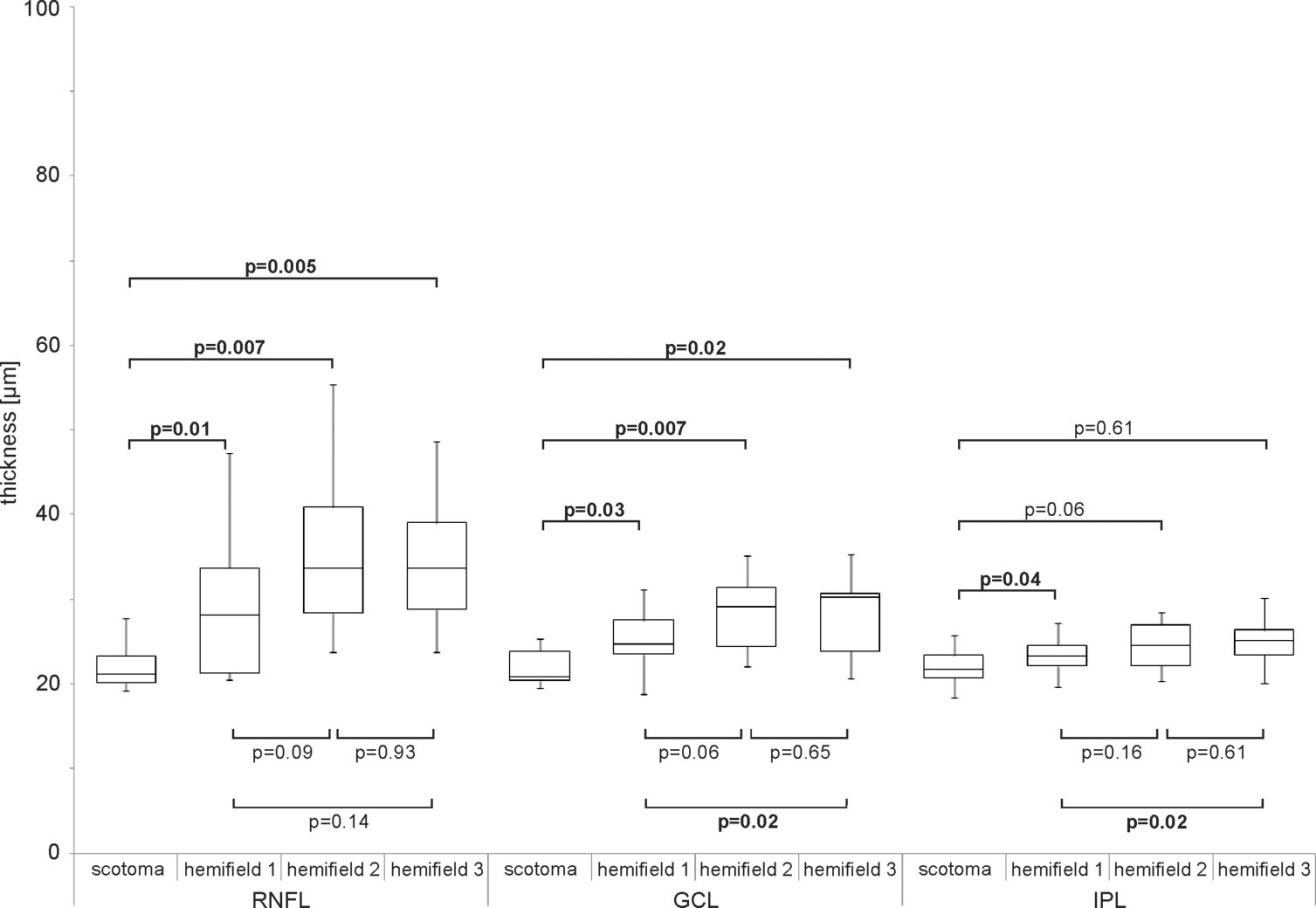
Investigated layers related to scotoma location. Box-and-whisker plots of the mean thickness of RNFL, GCL and IPL in glaucoma eyes investigated with reference to the localization of the absolute visual field scotoma. Scotoma: hemifield in which absolute visual field scotoma is located; hemifield 1: second hemifield of the eye with the absolute visual field scotoma; hemifield 2: corresponding hemifield to scotoma hemifield in contralateral eye; hemifield 3: second hemifield of the contralateral eye not corresponding to the scotoma hemifield.

A significant thinner mean RNFL thickness within the hemifield corresponding to the absolute visual field defect compared to the opposite hemifield of the same eye was found (p = 0.01) (Friedman-test and Wilcoxon-test as post-hoc analysis). Comparable differences have been found for GCL (p = 0.03) and IPL (p = 0.04) (Fig 3). Statistically significant RNFL thickness differences for the hemifield with the absolute visual field defect compared to both hemifields of the contralateral eye were also detectable (for details see Table 3 and Fig 3). This could also be demonstrated for GCL thickness measured for these particular hemifields. Comparable analyses concerning the hemifields of both eyes did not show differences of statistical significance for INL, OPL, ONL or ORL.

**Fig 4:**
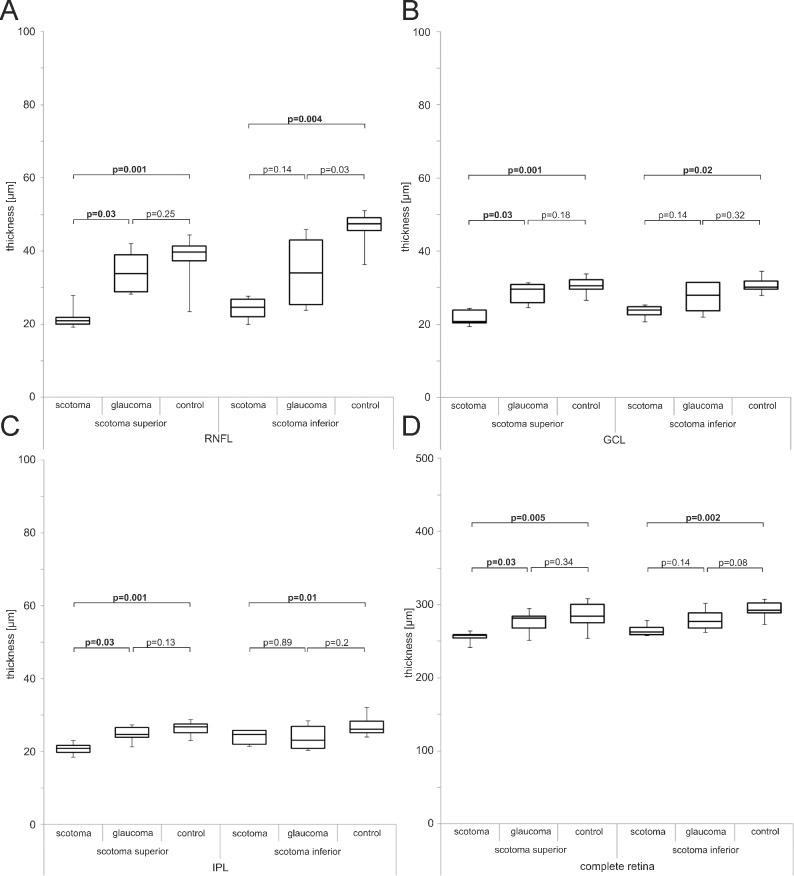
Hemifield comparison between glaucoma patients and control subjects. Box-and-whisker plots of the comparison of mean thickness of RNFL (A), GCL (B), IPL (C) and entire retina (D) between glaucoma group and control group related to the localization of the absolute visual field scotoma in superior or inferior hemifields.

A comparison between the mean thickness of each retinal layer of the hemifield corresponding to the position of the absolute visual field defect and the corresponding hemifield of the contralateral eye as well as the thickness of the hemifields of the healthy control groups was performed (Wilcoxon- and Mann-Whitney-U-tests). For this analysis glaucoma cases were matched depending on the localization of the absolute visual field defect i.e. if it was localized in the superior or inferior hemifields. The exact averaged measured thickness values for the different retinal layers included in this analysis can be taken from Table 4. Mean RNFL thickness of the hemifield corresponding to the absolute visual field scotoma in the glaucoma eyes was lower than in the corresponding hemifield of the contralateral eyes (p = 0.03) and also lower than the same hemifields in the healthy comparison group (p = 0.001) when the scotoma was localized in the superior hemifield. Coherent results were found, in the group of eyes where the absolute visual field defect lay in the inferior hemifield, which was only the case in four out of 15 glaucoma patients (Fig 4).

**Table 4:** Comparison of glaucomatous eyes and healthy control group. Mean thickness of the retinal layers and the entire retina as measured in the group of glaucoma patients and the healthy age-matched control group. Results are shown for the hemifields corresponding to the localization of the absolute visual field defect, corresponding hemifields of the contralateral eyes, and corresponding hemifields of the control group with respect to the localization of the scotoma in the superior or inferior half of the visual field.

	superior hemifield			inferior hemifield		
	absolute scotoma	corresponding hemifield in contralateral eye of glaucoma patients	healthy comparison group	absolute scotoma	corresponding hemifield in contralateral eye of glaucoma patients	healthy comparison group
**RNFL**	21.7 ± 3.0	34.4 ± 6.1	38.2 ± 5.7	24.3 ± 3.5	34.4 ±11.2	46.9 ±3.9
**GCL**	21.9 ± 2.1	28.6 ± 3.1	30.6 ± 2.4	23.5 ±2.0	27.4 ± 4.9	30.8 ± 2.1
**IPL**	20.7 ± 1.7	24.8 ± 2.2	26.3 ± 1.8	23.9 ± 2.1	23.9 ± 3.6	26.7 ± 2.3
**INL**	32.0 ± 2.5	31.1 ± 2.9	30.0 ± 1.9	32.6 ± 4.6	31.7 ±1.7	29.6 ± 3.3
**OPL**	26.4 ± 2.7	26.9 ± 2.9	26.1 ± 2.1	27.6 ± 1.9	27.4 ± 1.3	26.7 ± 2.3
**ONL**	53.6 ± 6.8	52.8 ± 8.1	59.6 ± 5.9	56.0 ± 6.4	54.2 ± 8.3	54.9 ± 5.2
**ORL**	79.0 ± 0.8	79.2 ± 1.2	78.2 ± 2.4	79.8 ± 3.2	79.3 ± 3.6	79.2 ± 2.8
**entire retinal thickness**	256 ± 7	276 ± 15	285 ± 19	265 ± 10	280 ± 18	294 ± 10

Thinner mean retinal thickness of GCL, IPL and the entire retina of the hemifield corresponding to the absolute field defect have been detected compared to the hemifields of the healthy comparison group. This relationship could be demonstrated for those cases containing field defects in the superior hemifield (GCL: p = 0.001; IPL: p = 0.001; complete retinal thickness: p = 0.005) and in those where the defect lay in the inferior hemifield (GCL: p = 0.02; IPL: p = 0.01; complete retinal thickness: p = 0.002). The results comparing layer thickness of those hemifields corresponding to the absolute visual field defects, the contralateral eyes of the glaucoma patients and the healthy comparison group can be taken from Fig 4.

Comparison of INL, OPL, ONL and ORL thickness between both eyes of the glaucoma patients and the healthy control group did not show any differences of statistical significance and are therefore omitted from graphical presentation. The absence of statistically significant results was the case when analyzing using the results gathered for the complete group of glaucomatous eyes as well as for the subgroups with the absolute visual field scotoma localized in the superior or inferior hemifields.

## Discussion

**Fig 5:**
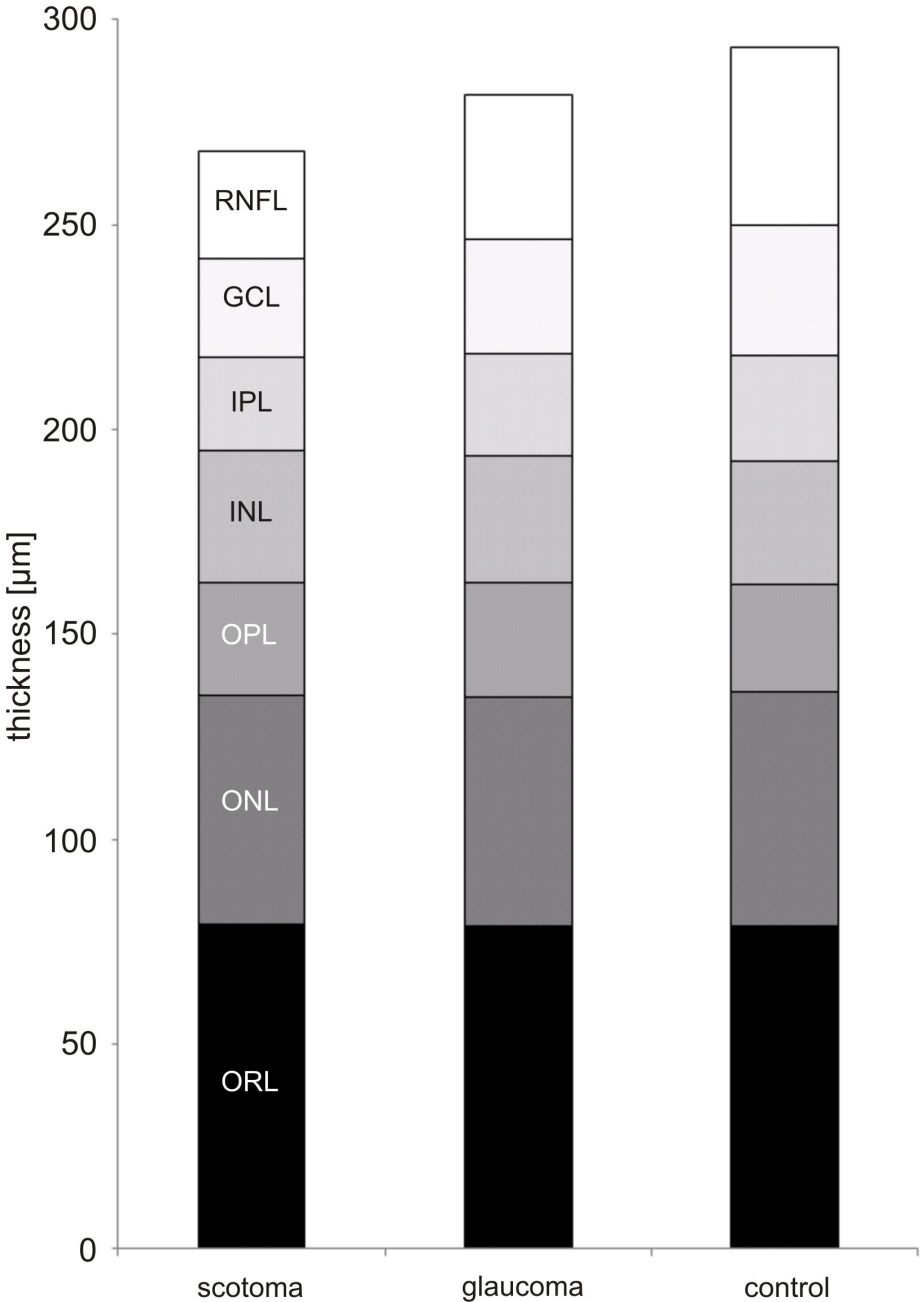
Summary of mean thickness values. Graphical representation of the seven retinal layers measured in the glaucoma eyes with the absolute visual field scotoma (scotoma), the contralateral eyes of the glaucoma patients (glaucoma) and the healthy control group depicted as stacked box plot which shows decreased layer thicknesses for RNFL, GCL and IPL but not for the four outer retinal layers.

The presented work indicates the impact of glaucoma on central retinal architecture in the studied population of patients affected by open-angle glaucoma in advanced disease stages with presence of unilateral single circumscribed absolute visual field scotomas. Glaucoma in advanced disease stages had a strong impact predominantly on the thickness of the inner retinal layers (RNFL, GCL and IPL). Present glaucoma resulted in a lower mean thickness of RNFL, GCL and IPL in those macular regions corresponding to the absolute visual field defects when compared to the less affected contralateral eye or a healthy age-matched control group. In contrast mean thickness of the outer retinal layers (OPL, ONL, ORL) remained unchanged. Fig 5 serves as a graphical summary of the aforementioned results concerning the thickness decrease most pronounced in the inner retinal layers in glaucoma.

Interestingly, the mean RNFL thickness in the healthy age-matched control group differed in superior compared to inferior hemifields. This could be due to the small sample size of glaucoma patients and controls but still led to different conclusions concerning the analysis of the presented findings. Firstly, it seems not reasonable to only compare mean thickness of superior and inferior hemifields in single glaucomatous eyes. Instead, the corresponding hemifields of both eyes of glaucoma patients should be compared to the corresponding hemifields of the healthy control group. Secondly, it seemed reasonable to analyze the mean thickness of other retinal layers than the RNFL to test if glaucoma in advanced disease stages has an impact. Thirdly, we chose to use the 8 x 8 grid array and not the default early treatment diabetic retinopathy grid used by other groups before for similar analyses, because of the missing strict separation of superior and inferior hemifields [25–27].

Previous studies reported of correlations between peripapillary RNFL and macular RNFL, GCL, IPL thickness which has not been studied in the presented work [13, 26]. Similar to the findings of Hood et al. we saw more patients with glaucomatous retinal thinning located in the inferior hemifields of the macula which corresponds to visual field defects above the horizontal midline [13]. The presented data strongly indicate the relation of decreased layer thickness in the inner retina with the main disease impact in advanced glaucoma stages [25]. Despite expectations, the data provided by SD-OCT and automatic retinal layer segmentation did not show any signs for retinal neurodegeneration upstream from the RGC [28]. However, the unaltered layer thickness does not necessarily mean that the number of cells in those layers is unchanged. Future investigations using advanced imaging methods like adaptive optics will provide in-vivo insights into the remodeling processes in the entire retinal architecture including the outer retina in glaucoma patients [29].

Common knowledge of morphological changes in glaucoma is related to the affected RGC. This cell and associated cell protrusions are located over the three inner retinal layers. The RGC axons form the RNFL, the cell bodies are found in the GCL and the RGC dendrites form synapses with bipolar cells and amacrine cells in the IPL. Previous studies reported about the possibility of measuring the RNFL, GCL and IPL thickness by using SD-OCT. Moreover, thickness decrease in those layers correlates roughly with the disease stages which are defined by presence and extend of visual field defects [30]. Moreover, macular IPL parameters were shown to be as good parameters for discriminating healthy from glaucomatous eyes as the thickness of the peripapillary RNFL [31].

Recent reports demonstrated the difference of RNFL and total macular thickness between the unaffected and the affected hemifield of single glaucomatous eyes detected by OCT [32, 33]. Related to the analysis settings used in this study, the thickness of all retinal layers in the worse affected hemifields were compared to the opposite hemifield of the same eye and additionally to both hemifields of the contralateral eye. Interestingly, the dataset revealed differences of retinal layers (RNFL, IPL, ONL and ORL respectively) in between superior and inferior hemifields in the healthy control group. In contrast, the other central retinal layers did not demonstrate these differences between superior and inferior hemifield in the healthy control group. These differences between mean thickness of superior and inferior hemifields of certain central retinal layers might be due to existent physiological disparities of retinal architecture or due to the small sample size. In glaucomatous eyes differences of retinal layer thickness in RNFL, GCL, IPL and entire retinal thickness have been found in the hemifields corresponding to the localization of the absolute visual field scotoma compared to the opposite hemifield. Within these layers, a difference of affected hemifields compared to healthy controls has also been found. Therefore, based on the superior-inferior RNFL-thickness-disparity the dataset indicates that the retinal layer measurements of GCL and total retinal thickness seem to be a more reliable candidate for the mentioned hemifield analysis in glaucoma by SD-OCT.

The presented method of measuring mean thickness of the inner retinal layers in different arrays of the macular region (Fig 2) can easily be included into the examination routine of glaucoma patients with regards to longitudinal and transversal patient management [34]. The main results of our study are that glaucoma predominantly affects the inner retinal layers which can be measured using OCT techniques and can help to differentiate between healthy and affected eyes. Agreement could already be demonstrated for circumferential peripapillary RNFL measurements using OCT and visual field testing introducing it to daily clinical routine years ago [35]. Additional analysis of both, the central retinal architecture and layer thickness in the macular region, together with the mentioned hemifield analysis could be utilized to test for plausibility of visual field scotomas. This would lead to clinical decision making gaining independence from psychophysical testing methods and the associated problems. The data set indicates the endorsed application of SD-OCT imaging techniques in the diagnosis and follow-up of glaucoma patients.

Future research efforts will be necessary to study the impact of different disease stages of glaucoma on SD-OCT measurements of retinal layer thickness and the presented hemifield analysis. Future developments may allow the analysis of retinal architecture peripherally from the central 10° where glaucomatous impact usually develops first. Studying patients in advanced disease stages together with the related remodeled macular architecture and individual layer thickness may limit the impact of the presented work. Less thickness differences of single retinal layers in moderate stages of the disease are very likely. Another limitation of this study is the small sample size which is mainly due to the strict inclusion criteria of glaucoma patients with an absolute visual field scotoma in the central 10° of the visual field in one eye and an unaltered visual field in the fellow eye. The reason for this was the design of the planned analysis where the fellow eye served as an internal control but in the end also showed marked glaucomatous disease impact. However, studies including larger cohorts of glaucoma patients may help to clarify the progression of retinal layer thinning in relation to the disease progression and to distinguish between healthy and diseased retina and to distinguish glaucomatous alterations from those of other retinal diseases (i.e. vascular or inflammatory) [25–27, 36]. Analysis of macular layer thickness using SD-OCT may also help in future glaucoma care in that it could exhibit susceptible retinal areas in which future development of deep scotomas is imminent which then should lead to intensify glaucoma treatment by lowering intraocular pressure.
